# Automatic sentence simplification system for Arabic Script Punjabi

**DOI:** 10.1371/journal.pone.0344915

**Published:** 2026-06-11

**Authors:** Tayyaba Shehzad, Sadaf Abdul Rauf, Saleha Nazeer, Ali Daud, Hussain Dawood

**Affiliations:** 1 Department of Computer Science, Iqra University, Islamabad, Pakistan; 2 Department of Computer Science, International Islamic University Islamabad, Pakistan; 3 Department of French Language and Literature, University of the Punjab, Lahore, Pakistan; 4 Faculty of Resilience, Rabdan Academy, Abu Dhabi, UAE; 5 School of Computing, Horizon University College, Ajman, UAE; 6 Jadara University Research Center, Jadara University, Jordan; Southwest University, CHINA

## Abstract

In the domain of language simplification, creating aligned monolingual parallel datasets tailored to specific linguistic dialects is a significant endeavor. This pursuit, introduces the pioneering Punjabi Simplification (PUSIM) corpus, which focuses on the Shahmukhi dialect. Shahmukhi, one of the two prominent dialects of Punjabi, serves as the foundation for this corpus development. This study employs a hybrid approach that ensures the comprehensive assessment of simplification outcomes. The detailed process of simplification underwent thorough examination, aiming to transform complex sentences into simpler ones by enhancing writing clarity and vocabulary. To quantify the quality and readability of simplified texts, automated readability assessments were conducted using well-established text readability metrics, a significant SARI score of 45.3, attested to the high quality of simplification approach. The unique aspect of this work lies in its focus on the Shahmukhi dialect, addressing a linguistic facet that had previously received limited attention in natural language processing. It is anticipated that this dataset will pave the way for further exploration and research, offering novel possibilities for leveraging automated simplification techniques in the realm of Shahmukhi Punjabi language processing.

## 1. Introduction

Comprehension of complex sentences creates an immense hurdle for new language learners, particularly those who lack skill in the language, struggle with reading, and people with cognitive disabilities [1–5]. Having simplified text can greatly facilitate such individuals, where sentence simplification is a strategy that breaks down difficult phrases into their simplest form to improve readability [6–8]. The importance of simplicity was emphasized in the late 1990s by initiatives that stressed conveying essential information in easily understandable manner and proposed suggestions to enhance the clarity of communication [9,10].

It has been demonstrated that easily comprehensible writing provides advantages to readers, especially individuals with lower levels of literacy [11], language learners [12,13]. It also aids individuals diagnosed with dyslexia [14], autism [15], aphasia [16]. Individuals with learning difficulties tend to perceive reduced sentences as more comprehensible [17,18]. Text simplification also has wide applications in Natural Language Processing(NLP) tasks as a prepossessing step, for example,to improve the performance of parsers [19], summarizers [20], and semantic role labelers [21].

Most of the studies on simplification have concentrated on English where source texts and related simplified texts are available and can be automatically aligned such as English Wikipedia [22]. This remains and unexplored area for most languages due to unavailability of simplification corpora.

To facilitate the study of complexity and simplicity parameters across various languages, it is essential to possess a corpus comprising pairs of sentences along with their simplified versions. Such corpora, known as sentence-aligned simplification parallel corpora, have been compiled for multiple languages. For instance, in English, resources like the Simple Wikipedia corpus PKWP [23], Newsela [24], Onestop [25], and ASSET [26] have been established. Similar efforts have created resources for Spanish (An-Cora [27]), Italian (ERNESTA [18]), French (CLEAR [28] and Alector [29]), and other languages. The development of such simplification corpora, containing a sufficient number of complex sentences and their corresponding simplified versions, is a critical first step. Unfortunately, there is currently a lack of study on simplifying sentences in low-resource languages.

Punjabi is an Indo-Aryan language that is widely spoken in Pakistan and India (https://simple.wikipedia.org/wiki/Punjabi_language). The primary focus of our research is Punjabi language written using the Shahmukhi (شاہ مُکھی) script. Despite the huge number of speakers (https://en.wikipedia.org/wiki/Punjabi_language), this particular variation has received relatively less attention, particularly within the fields of natural language processing and sentence simplification. Punjabi is one of the world’s top ten languages and is written using “Gurmukhi” and “Shahmukhi” scripts based on geographical location and spoken in a variety of dialects (https://en.wikipedia.org/wiki/Punjabi_dialects_and_languages) (Accessed on: 15 september 2025). The language’s large user base gives it considerable weight in the field of natural language processing.pujabi text book syllabus is available at (https://pctb.punjab.gov.pk/) (Accessed on: 1-december-2025) which also shows the importance of this language. To the best of our knowledge, the only prior work on Punjabi text simplification is by [30], which focuses on the Gurmukhi script. While their work is highly relevant as it addresses the same language, the significant orthographic, morphological, and lexical differences between the Gurmukhi and Shahmukhi scripts present unique challenges that necessitate a separate investigation. This work is, therefore, the first to address simplification for the Shahmukhi script.

The foundation of our work is a manually crafted parallel corpus. It consists of **1,400** original complex sentences, each manually simplified to create a high-quality reference simplification, which we designate as **S1**. This gives us **1,400 {Complex, S1}** core pairs.

To augment this data and introduce syntactic diversity, we generated three additional simplified variants for each complex sentence. Using the manually created S1 simplifications as a base, we applied a set of rules for three primary operations: lexical substitution, deletion, and insertion. This process produced three new versions for each sentence: **S2, S3, and S4**.

Therefore, for every one of the 1,400 complex sentences, we have a **quadruplet** of four simplified versions ({S1, S2, S3, S4}), resulting in a total of **1,400** × **4 = 5,600 simplified sentences**. The entire corpus comprises **1,400 complex sentences aligned with 5,600 simplified sentences**, organized into 1,400 quadruplets. An example quadruplet is shown in Table 4.

The contribution can be summarized as follows:

- Creation of a corpus named PUSIM for the evaluation of Punjabi sentence simplification model,will be followed by its public release at https://github.com/sabdul111/Punjabi-Simplification-Corpusit will be the first open source simplification dataset in Punjabi language.
- Corpus contains manual simplification with three different versions of per original sentence.

This paper is organized as follows: Section presents a brief overview of sentence simplification. Section 2 describes our proposed approach and system architecture and begins with the process of corpus creation, followed by the main steps involved in building the PUSIM corpus. Two types of evaluations are then conducted: automatic evaluation, discussed in Section 5, and human evaluation, presented in Section 3. A readability analysis is also performed to further assess the results.

Section 1 reviews the related work, and finally, Section 7 concludes the paper with a general discussion and future directions.

## 2. Related work

Various methods have been used to simplify sentences in the past, including rules that were hand-designed [12,13,31–33] using synonyms and paraphrases for syntactic and lexical simplification [34–36], husing embeddings [37] and viewing simplification as a monolingual machine translation problem where operations are learned from examples of complex-simple sentence pairs [23,38–40]. Many unsupervised techniques have been suggested in recent research to train simplification models without a labeled simplification corpus, which is a solution to the problem of low-resource languages and the shortage of parallel SS corpus [41–44]. MUSS [45] achieves impressive results in Spanish and French, surpassing even the supervised state-of-the-art. Many subsequent studies have employed this approach in their attempts to simplify English sentences. These studies have utilised various methods such as phrase-based machine translation [46] and syntax-based machine translation [23,31] or neural machine translation [22,47]. According to a comprehensive manual evaluation, the neural machine translation (MT) model developed by [47] demonstrates superior performance compared to the phrase-based MT model proposed by [40], as well as the syntax-based MT model introduced by [23,31,47].

### 2.1. Simplification dataset in multiple languages

[24] made the Newsela corpus, which is the first English simplification corpus created by humans. This corpus contains articles that are rewritten at four different levels to make them easier to read for children of different ages. Similarly, [48] created an Italian simplification corpus using three levels of simplification: local coherence, global coherence, and lexical/syntactic changes. They simplified the syntax and vocabulary by rearranging words, adding, splitting, merging, transforming, and deleting them. These simplification methods were also used by [49] and [50]. There exists a lot of supervised training corpora [22,24] and a high quality dataset for English Sentence simplification [1,31,51]. On the other hand, there is a great deal of interest in and use for automatic SS systems written in other widely used languages. However, there is a growing demand for automatic sentence simplification systems in popular languages other than English. Researchers have tried to explore simplification in these languages, such as [8,52], and [42]. But they face a challenge because there aren’t enough parallel corpora available.

Recently, some projects have focused on creating sentence simplification datasets in other languages that don’t have many resources. These projects include works by [48,53,54]. These datasets help in developing multilingual sentence simplification techniques [55] like ALECTOR [29], Simpitiki [49], and the Spanish part of Newsela [24].

The complex linguistic structure of Shahmukhi Punjabi, which is written in a script different from its Indian cousin Gurmukhi, has not received substantial attention in computational linguistics. Compared to the rule-based morphological approach of [30] Punjabi sentence simplification through a rule-based linguistic and morphological framework, the present study adopts a dataset-driven and dialect-specific perspective. [30] focus on participial complex sentences, where simplification relies on the identification of non-finite verb suffixes (e.g., “ਦਿਆਂ/ਇਆਂ”) to detect dependent clauses and subsequently convert them into independent clauses using explicit linguistic rules. Their methodology combines lexical simplification, syntactic clause segmentation, and content reduction, achieving high system performance with precision, recall, and F-measure exceeding 93%. In contrast, the current work does not constrain simplification to a single sentence subtype; instead, it introduces the Punjabi Simplification (PUSIM) corpus, developed specifically for the Shahmukhi dialect, a largely underexplored linguistic variety in NLP. Rather than relying on handcrafted linguistic rules, the proposed approach emphasizes parallel monolingual corpus creation and automated readability assessment, which simplifies participial Punjabi sentences through clause boundary identification and non-finite to finite verb transformation. The present work adopts a dialect-focused and corpus-driven perspective. Additionally, due to Shahmukhi Punjabi’s linguistic and cultural specificity, its historical evolution shapes its syntactic and lexical properties, making it distinct from standard NLP pipelines [33]. Although several efforts have been made to enhance low-resource languages [33,56]. Recent efforts have resulted in the creation of annotated Shahmukhi Punjabi corpora; these resources are designed for linguistic analysis tasks other than text simplification. For example, the dataset presented in [57] focuses on Named Entity Recognition (NER) and is carefully annotated for entities such as persons, locations, and organizations, primarily supporting sequence labeling and information extraction tasks. Similarly, the available [58]Shahmukhi POS-tagged and named-entity datasets are structured with token-level annotations and task-specific objectives such as part-of-speech tagging or entity classification, rather than sentence-level semantic rewriting. However, to our knowledge, no effort has been made to compile a dataset specifically for Punjabi SS. The simplification scheme defined in the research literature is followed, and the most frequent evaluation metrics are used to set the ground work for future study on Punjabi.

## 3. Corpus creation

The study utilized monolingual resources from the EMILLE Corpus (Baker et al., 2008), distributed by the European Language Resource Association (ELRA). The Punjabi component of this corpus, which comprises traditional narratives and stories, was selected for this work. The raw corpus contains 375,366 sentences.

A subset of 1,400 sentences was curated from this larger corpus for the purposes of this study. The sentence selection procedure was designed to align the data with the target audience—individuals with a basic level of understanding and Grade 12 to Grade 16 students. The exclusion criteria were as follows:

Temporal Relevance: Sentences containing vocabulary, idioms, or cultural references that are largely archaic and no longer in common use in modern, everyday Punjabi were excluded. This ensures the study’s relevance to contemporary language learners.

Lexical Modernity: Preference was given to sentences where words appear in their current, evolved morphological forms, rather than their historical or literary variants.

This process resulted in a manageable and relevant dataset focused on high-frequency, modern Punjabi usage, upon which the subsequent simplification tasks were performed.

Motivation for Choosing the EMILLE Corpus:A primary motivation for selecting the EMILLE Corpus was its status as a foundational, broad-coverage resource for languages, providing a robust and diverse starting point for linguistic research. While the corpus contains historical and literary material, its size and variety allowed for the intentional selection of a modern-facing subset. This approach of curating a relevant sub-corpus from a larger, more general resource is a established practice in NLP, allowing us to leverage the corpus’s strengths (scale, authenticity) while mitigating its limitations (temporal spread) for our specific task of text simplification.

### 3.1. Simplification scheme

In order to enhance the ease of understanding without altering the essential information, manual simplification is used to ensure correctness. The simplification methodology is consistent with previous works [26,43,59,60] and [28,50]. As we have adopted the same core simplification operations that were formally established and applied in the referenced literature. This means we employed identical linguistic strategies—such as rephrasing, insertion, deletion, and reordering—following the definitions, guidelines, and practical implementations described in these prior studies. By applying these well-recognized operations to the Punjabi language, we ensured that our simplification process aligns with internationally accepted methodologies.

First version of the corpus was mainly based on rephrasing, insertion, deletion, substitution, merging and reordering. The comparison of these operations is presented in Table 1. The 3 subsequent versions were created using lexical and phrase substitution.

**Table 1:** Simplification operations were applied in different corpora.

Corpus	Substitution	Deletion	Insertion	splitting	Merging	Reordering
Turk (2006)	yes	yes	yes	–	yes	–
PKWP (2015)	yes	yes	yes	yes	–	yes.
SIMPITIKI(2016)	yes	yes	yes	yes	yes	yes
SIMPA (2018)	yes	yes	_	yes	_	yes
ASSET (2020)	yes	yes	yes	–	_	yes
SIMUR(2021)	yes	yes	yes	yes	yes	
CSS (2023)	–	yes	yes	yes	yes	
PUSIM (OURS) 2026	yes	yes	yes	yes	yes	yes

Two annotators worked on simplification which took almost 500 man hours. Both were Punjabi natives with fluent proficiency with one being a linguist and Punjabi expert. The steps are detailed below with relevant examples, an overview of the procedure is depicted in Figurefig:simplification-operations. One annotator performed the primary simplification work on the corpus while the second annotator carried out a thorough, independent review. Each simplified sentence was checked repeatedly in an iterative manner to ensure accuracy and consistency: the reviewer inspected every sentence, suggested corrections where necessary, and the pair discussed and resolved any disagreements. This careful, repeat-check process served as an internal quality-assurance step to guarantee that the final simplified sentences are both faithful to the originals and easy to understand. They are not paid. The existing simplification corpora have been developed for several languages such as English, Italian, and Turkish and Chinese In contrast, PUSIM is designed for Punjabi, addressing the lack of simplification resources for low-resource languages.

### 3.2. Lexical and phrase substitution

**Fig 1:**
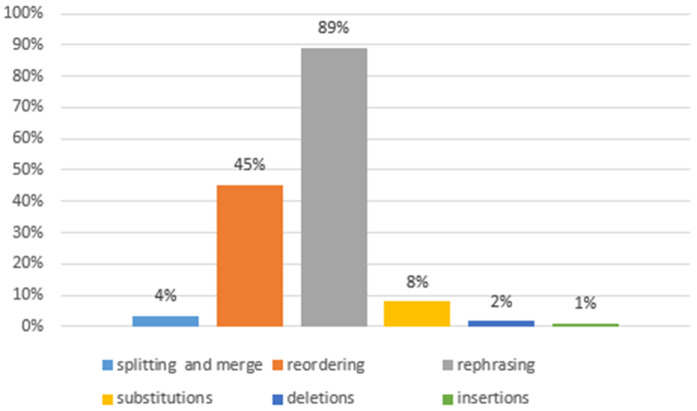
Simplification operations of manual corpora.

Lexical substitution is a tool for simplifying sentences by replacing difficult words with easier equivalents without altering the intended meaning [6]. For instance تھاں in Punjabi means $\ddot{p}$lace” which is replaced with its simpler alternative جگہ, which is easier to understand. In phrase level substitution a complex phrase is replaced with a correspondingly easier phrase for example " بڑی دھیان گوچری اے” which means “to focus” is replaced with " بڑی غور کرن والی اے” “focus”. The guidelines of these decisions were rooted in the principal of modern frequency usage in punjabi. This approach is consistent with the manual simplification paradigm where annotators apply linguistic expertise to judge complexity as shown in the guidelines 6 The context is properly followed so that the meaning of sentence remains unaltered. 89% of our sentences underwent as shown in Fig 1.

#### 3.2.1. Complex:Simple Lexicon.

A by-product of lexical and phrase substitution process was a 630 entry lexicon containing 390 word level and 254 phrase level entries. Some examples are shown in Table 2 and 3.

**Table 2:** Some examples from the complex:simple lexicon.

Phrase level Substitutions		
Get carried away while talking	" دھن وچ مگن”	“گلاں وِچ مست”
Very attentive	" بڑی دھیان گوچری اے”	" بڑی غور کرن والی اے”
In your surroundings	“اپنے دوالے”	" “اپنے ارد گرد "
**Word level**	**Substitutions**	
News or update	" اُگھ سُگھ”	" خیر خبر”
place	تھاں	جگہ
Ask	آکھدے	کہندے

**Table 3:** Frequency-based Lexicon Sample.

Word	Simpler Alternative	Frequency
تھاں	جگہ	8
آکھدے	کہندے	16
" اُگھ سُگھ”	" خیر خبر”	22

few lexicon with the word level frequencies are mentioned.

### 3.3. Reordering

This process invollexicon ves switching around the sequence of words or phrases, such as rearranging the clauses of a sentence to make it more concise. Reordering was the 2^*nd*^ largest operation, with 45% of reordered sentences in the simplified corpus. The first sentence shows complete sentences reordering, whereas the second sentence demonstrates reordering at sentence tail only.

*Original* = انتظامیہ والیاں آکھیا ایہنوں گولی مار دیو بس اے سندیاں ای ونجھل انج رون لگ پیا جیویں کوئی چھوٹا جہیا بچہ ہووے
*Simplified* انتظامیہ والیاں آکھیا ایہنوں گولی مار دیو س اے سندیاں ای ونجھل بچياں دی طرح رون لگ پيا

When the management asked him to shoot, vanjal started crying like a child

*Original* دسيا کے مائی نیں اوہناں نو
*Simplified* مائی نیں اوہناں نو دسيا

women told them

### 3.4. Merging

Merging is a technique used to combine two or more phrases into a single, easier-to-understand one. Typically, this is done by switching around a few words in a phrase or by moving a sentence around. Joining two independent sentences using coordinating conjunction such as “and” or “but” is also a merging technique. PUSIM contains 4% merging operations. Some example sentences are shown below:

*Merge Original* اوہنوں سُجھ گئی کہ ہووے نہ ہووے ایہہ ہنس ای نیں، جیہڑے اوہدے وِیر نوں چُک کے لَے گئے نیں
*Simplified* اوہ سمجھ گی کے ہنس ای اوہدے وِیر نوں چُک کے لَے گئے نیں
She realized that the swans had taken her brother away
*Merge Original* سیکریٹری ہکا بکا رہ گیا پئی ایہہ کیہو جیہا گویا اے، جیہڑا پیسے وی نہیں لیندا
*Simplified*
سیکریٹری بڑا حیران ہویا کے ایہہ کیہو جیہا گویا اے جو پیسے نہیں لیندا

The secretary was greatly surprised, wondering what this is, as if he is someone who doesn’t take bribe.

### 3.5. Insertion

Insertion is a fundamental operation that involves adding an element. This addition occurs at a designated position within the structure, ensuring that the overall organization and sequence of elements too remain intact while ensuring meaning preservation. It is possible for the simplified statement to be longer than the original since it might include explanations or clarifying terms. PUSIM has 1% insertion operations. Note that this count includes only the standalone insertion operations the insertion operations that were performed in rephrasing section (2.1) are not included in this count. An example sentence is shown below:

*Original* تیری کھوج وِچ عقل دے کھمب جھڑ گئے
*Simplified* تیری تلاش وِچ عقل تے سوچ موک گي
My intellect and thinking will end in your search

To simplify this sentence more precisely, The words are added likeتے عقل which means “intelligence” is inserted and کھوج which means “search” is replaced by تلاش.

### 3.6. Deletion

Deletion involves removing redundant information from a sentence while ensuring meaning preservation. In our corpus overall word deletion ratio is 2%. An example sentence is shown below where unnecessary words like تے کوئی “and some” are deleted to simplify.

*Original* تے کوئی کجھ وچوں اک نیں آواز لائی
*Simplified* کسے نیں وچوں آواز لائی

Someone called out from the middle

### 3.7. Versions of simplification

Three additional versions of the simplification corpus were created using the simplified corpus as a base. These simplified versions were created by adding, removing, or changing words in the original simplification. Each sentence now has four different simplified versions. These are reffered to as Expanded Simplification Variants (ESV). Table 4 shows the four variants], S1 denotes first level simplification, and S2 til S3 denote the next simplifications.

**Table 4:** Versions simplification: The first sentence S1 is the main simplified sentence, and the next three, S2, S3, and S4, are lexically simplified versions of it.

Name	Original Sentence	Simplified Versions
O	جیہڑا انتاں دا شرارتی سی	
S1		جیہڑا بہت شرارتی سی
S2		شرارتی جیہڑا بہت سی
S3		جیہڑا بہت مستا سی
S4		جیہڑا شرارتی سی بڑا
English	The one who is very naughty.	

We rephrase the word انتاں دا with بہت and in the next sentence, the main word شرارتی is changed with مستا so by reordering, rephrasing,and deletionwe have created different variants to choose the best possible version of simplification (Table 5).

**Table 5:** S1 is the syntactic simplification, whereas S2 - S4 denote the lexical simplifications of S1.

	Complex	S1	S2	S3	S4
Average sentence length	12.6	12.1	12.2	11.8	11.0
Lexical diversity	0.3	0.2	0.2	0.2	0.2
Total number of words	17653	16973	17059	16618	16413

Fig 1 and Table 5 show the percentage of each simplification operation on the x-axes applied during the syntactic simplification S1. Rephrasing was the most significant operation with 89% of sentences being rephrased, this is in agreement with previous works, e.g. [46] used 65% of rephrasing operation for English simplification. Reordering was the second most significant operation with 45% sentences. Only 4 % of sentences employed splitting and merging. Punjabi tends to require more structural adjustments during simplification due to morphological complexity. Many original sentences contained cultural idioms and compounding that required rephrasing rather than simple lexical or deletion operations. Therefore, the higher rephrasing percentage reflects linguistic characteristics of Punjabi rather than inconsistency. The high rephrasing percentage also shows how much careful effort we put into manually simplifying the corpus. Punjabi sentences often cannot be simplified by just replacing a word—they need to be rewritten for clarity. This required our annotators to spend more time and attention on every sentence.

## 4. Human evaluation

Human evaluation was done by four graduate native Punjabi speakers on 30 randomly selected sentences. The sentences were evaluated for adequacy, fluency, and simplicity. The annotators were asked to rank the sentence pairs based on the three parameters shown in Table 6. Q1 measures simplicity, Q2 is based on the adequacy, which measures meaning preservation, and Q3 measures fluency of the sentences. Ranking was done using Likert scale [61], the participants were asked to rank the sentence in the range of 1–5. Possible rankings were: 1 for “strongly disagree,” 2 for “disagree”, 3 for “average,” 4 for “agree” and 5 is for “strongly agree.” The pairwise inter-annotator reliability for the 30 phrases evaluated by the four evaluators. However, our sample size is consistent with established practice in human evaluation for text simplification, where researchers commonly use 20–50 sentences for subjective quality assessments such as simplicity, fluency, and adequacy. Several prior works on text simplification related NLP tasks (e.g., readability evaluation, summarization quality judgments, and controlled generation) have used small but carefully selected random samples to assess human-perceived quality. These studies argue that human evaluation is costly, time-intensive, and requires expert annotators, therefore smaller but representative samples are widely accepted when:Annotators are expert-level (as in our case: four graduate native Punjabi speakers),

**Table 6:** Guidelines for human evaluation.

Rule	Guide lines for simple form	
Q1	Simplicity	Reference Sentence is understandable.
Q2	Adequacy	The original meaning of sentence is preserved.
Q3	Fluency	The sentence is grammatically correct.

Sampling is random, ensuring no selection bias, and Evaluation focuses on subjective dimensions rather than model-level statistical performance. Fleiss kappa score, which is used commonly used to measure inter-annotator agreement for more than two annotators was calculated on the corpus.

### 4.1. Fleiss kappa: Inter-annotator agreement scores

Fleiss kappa [62] is a statistical measure used to assess the degree of agreement among multiple raters when assigning categorical ratings to items or classifying items. It is an extension of Cohen’s kappa [63], which is normally used to assess the agreement between two raters.

Generally, for Fleiss kappa scores greater than 0.75 indicate excellent agreement, values between 0.75 and 0.40 indicate good to fair agreement, and values less than 0.40 indicate poor agreement (https://www.datanovia.com/en/blog/kappa-coefficient-interpretation/). Table 7 shows our Fleiss’ kappa scores for different aspects, i.e., fluency, adequacy, and simplicity based on the super scale and lower scale. All aspects show excellent agreement on the super scale and a good agreement on the lower scale, on average it is excellent agreement on both scales 0.89, 0.75. These scores indicate that the PUSIM corpus demonstrates reasonable consistency among raters, especially considering the complexity of evaluating fluency, adequacy, and simplicity. Super Scale (Positive ratings): 4–5

**Table 7:** Inter-annotator agreement scores using Fleiss Kappa, the standard deviation are given in brackets.

Fluency	Adequacy	Simplicity	Average
Super scale 0.90(0.26)	0.88(0.19)	0.90(0.30)	0.89(0.36)
Lower scale 0.64(0.27)	0.69(0.15)	0.63(0.34)	0.75(0.27)

Lower Scale (Non-positive ratings): 1–3

This binary separation follows standard practice in human evaluation studies where upper Likert levels represent high-quality outputs and lower/mixed ratings are grouped together for contrastive analysis.

## 5. Simplicity and readability

Readability metrics measure the degree, that how easy or hard is a text to understand [64]. These metrics use mathematical formulas based on the factors associated with the lexical content of the text. These factors include the number of characters, syllables, words, sentences, difficult words, average sentence length, average word length and average difficult words etc.

Flesch Reading Ease (FRE) [65] and Flesch-Kincaid Grade Level (FKGL) [66], SMOG [67] and Automated Readability Index (ARI) [68] were used to grade PUSIM simplifications. Since, no prior work exists on readability parameters for Punjabi, conventional readability parameters tweaked according to lexical features of Punjabi were chosen. Flesch Reading Ease scores range from 0 to 100. If the score is high, the text is easy to read. If it’s low, the text is harder to understand. Table 8 outlines the readability ranges for with each metric indicating average or skilled levels.

**Table 8:** Score range for readability metrics.

Metric	Score	Level
FRE	0–30	Skilled
	60–70	Average
	90–100	Basic
FKGL	13–18	Skilled
	7–12	Average
	1–6	Basic
ARI	13–18	Skilled
	7–12	Average
	1–6	Basic
SMOG	111–240	Skilled
	13–110	Average
	1–12	Basic
LIX	55+	Difficult
	50–55	Skilled
	40–45	Average
	30–35	Basic
CLI	11+	Difficult
	8–11	Skilled
	5–8	Average
	1–5	Basic

### 5.1. Readability metrics

The metrics we selected for analysis FKGL, ARI, and SMOG are directly related to complexity. A higher score indicates more complexity, while a lower score means the text is simpler. However, with FRE, it’s the opposite: a higher score means the text is simpler. The goal was to get a relative, comparative score. We used the exact same formula to calculate a score for both the original complex text and our new simplified text. Even with this simple method, our simplified corpus consistently showed a better (higher) readability score than the original complex text. In Table 9 we have some interesting observations. The lowest score on FKGL [66] indicates that even a difference of small points is important in categorizing the level of text.

**Table 9:** Scores of original and Simplified sentences against FKGL, FRE, ARI, SMOG, LIX and Coleman-Liau.

	FKGL	FRE	ARI	SMOG	LIX	Coleman-Liau
Original	3.19	94.5	7.0	3.0	60	4.9
Simplified	2.3	100	5.3	3.1	53	3.7

The ARI (Automated Readability Index) and LIX scores indicate notable improvements in the readability of the simplified corpus compared to the original text. The main formula of LIX score is depend on the number of difficult words. The method was developed in 1968 by Swedish linguist and is widely used in European countries to assess the readability of documents. We calculate the difficult word as the word with greater than or equal to 5 characters is considered difficult. After applying the formula,


$$\begin{array}{c} \text{LIX Score}=\frac{W}{S}+100\times \frac{WD}{W} \end{array}$$ (1)

Where, *W* = Number of words. *S* = Number of sentences. *WD* = Number of difficult words. The LIX score for the original corpus was 60, classifying it as “very difficult.” After simplification, the score reduced to 53, categorizing it in the “skilled” range. This shift reflects a reduction in the number of difficult words and improved overall readability. The ARI score for the original corpus was 7.0, which places it in the “average” complexity range. After simplification, the score improved to 5.3, placing it in the “basic” range.

While improvements were observed in all metrics, the scores of the simplified corpus remain within their respective readability ranges, validating the enhanced accessibility of the text. The FKGL score indicates the minimum reading level necessary to comprehend the content. But this metric can easily count the number of English words and syllables, and we apply the formula to our Punjabi corpus to get the results. Even though this metric is generally for English, The simplified corpus shows better results simplying that the readability is improved. FRE [65] indicates a higher score means the text is simpler. To calculate the FRE score, we need words, sentences, syllables, and characters. We made adjustments to the syllable counting algorithm. We defined a syllable as a unit of sound containing a vowel. Our algorithm scans each word and counts a syllable for every occurrence of the following core vowel characters: The syllable count in Punjabi Shahmukhi is [”ا” ,“آ” ,“و” ,“ؤ” ,“ی” ,“ے” ,“ہ”]. This adjustment improves the dependability of syllable-counting readability measures which shows the original corpus has 94.5 and in case of simplified corpus its 100. The SMOG score changed minimally from 3.0 (original) to 3.1 (simplified). While this indicates only a minor difference, the metric remains somewhat unreliable for Punjabi due to its reliance on polysyllabic word counts.

We made a number of adjustments to how we computed readability ratings for our Punjabi corpus so that we could better reflect the specifics of that language. After making these adjustments, we examined our simplified corpus for readability metrics and found that it was simplified.

The following lexical features are to be extracted from the given text for several readability formulas.

- Number of characters in the text.
- Number of syllables in a text.
- Number of words in the text.
- Number of sentences in the text.
- Number of difficult words. It is different for every language.
- Average sentence length. Calculated using the following formula.
  $$\begin{array}{c} \text{Average Sentence Length}=\frac{\text{Number of Sentences in Text}}{\text{Number of Words in Text}} \end{array}$$ (2)
- Average word length. Calculated using the following formula.
  $$\begin{array}{c} \text{Average Word Length}=\frac{\text{Number of Letters in Text}}{\text{Number of Words in Text}} \end{array}$$ (3)
- Average number of difficult words. Calculated using the following formula.
  $$\begin{array}{c} \text{Avg No of Difficult Words}=\frac{\text{No of Difficult Words in Text}}{\text{No of Words in Text}} \end{array}$$ (4)

## 6. Automatic text simplification model

Phrase-based MT [69] has been a popular choice to develop Automatic Text Simplification (ATS) systems, especially when small amounts of simplification corpus are available [34,38,39]. Recent Neural text simplification [13,22,47,70,71] methods have significantly outperformed the SMT-based systems, but they require huge amount of corpora, which makes the SMT the most suitable choice for our systems. Multilingual sequence-to-sequence models like mBART, mT5, NLLB-2005, and mmT517 indeed support Shahmukhi and offer state-of-the-art performance for translation tasks. These models excel in zero-shot cross-lingual transfer (e.g., mT5 reduces source language hallucination from 7% to 9% in zero-shot settings) [72]. Massive multilingual coverage (e.g., NLLB-200 supports 200 languages, including 55 African languages) [73]. Denoising pre-training (e.g., mBART improves low-resource MT by up to 12 BLEU points) [74]. However, these models require large-scale corpora and substantial computational resources for training and fine-tuning. For example, NLLB-200 has 54 parameters and was trained on a supercluster [75]. Recent multilingual sequence-to-sequence models such as mBART, mT5, NLLB-2005, and mmT5 indeed support Shahmukhi and have achieved state-of-the-art performance in translation-related tasks. These models perform particularly well in zero-shot cross-lingual transfer; for example, mT5 reduces source-language hallucination from 7% to 9% in zero-shot settings [72]. They also provide massive multilingual coverage (e.g., NLLB-200 supports around 200 languages, including 55 African languages [73]), while denoising pre-training strategies such as those used in mBART have been shown to improve low-resource machine translation by up to 12 BLEU points [74]. However, such models typically require large-scale corpora and significant computational resources for both training and fine-tuning. For instance, NLLB-200 consists of approximately 54B parameters and was trained on a supercluster [75]. In comparison, Phrase-Based Statistical Machine Translation (PBSMT), particularly Moses, remains more suitable for low-resource Automated Text Simplification (ATS) tasks. These systems are more data-efficient and can perform reasonably well even with limited parallel data. In addition, data selection techniques can help optimize small datasets by extracting pseudo in-domain sentences. Furthermore, SMT-based ATS systems have demonstrated effectiveness for low-resource languages in prior studies [76], making them a practical and appropriate choice for Punjabi text simplification in resource-constrained scenarios.

We developed standard phrase-based Statistical Machine Translation (SMT) systems using the default configurations of the Moses toolkit [77]. For language modeling, we used a 5-gram KenLM model [78]. Language models for each system were trained on the target side of the parallel corpus. Word alignment was performed using Giza++ [79] with the widely adopted grow-diag-final-and-symmetrization technique. Maximum sentence length of 100 for word alignment and a distortion limit of 6 with a 100-best list for reordering were used. We used msd-bidirectional-fe lexical reordering model with a phrase table limit of 5.

Train, development and test sets were split in 80:10:10 ratio. SARI score was computed for 2 different test sets. The *self test set*has only a single reference from the test split of the same system. The *combined test set* contains multiple references, where four reference simplifications are given for each sentence. SARI supports multiple references, enabling a better evaluation of simplification scores. Parameters in Moses were fine-tuned on development data with the Minimum Error Rate Training (MERT) tool.

### 6.1. Evaluation metrics

We evaluate the performance of the system using two key metrics: BLEU and SARI scores, each serving distinct purposes. BLEU (Bilingual Evaluation Understudy) [80] is widely used in machine translation to evaluate the quality of the output. BLEU measures the adequacy by reflecting how closely the output aligns with reference translations. It calculates the similarity between the system-generated text and one or more reference sentences by measuring overlapping n-grams, with adjustments for brevity to prevent overly short outputs. This metric has shown a high correlation with human judgments on grammatical accuracy and, to a lesser extent, on content preservation.

In contrast, SARI introduced by [24] is designed to evaluate text simplification and emphasizes content addition, deletion, and retention relative to the original text. SARI compares the system output not only with reference sentences but also with the original input, rewarding appropriate additions from the reference that were absent in the input. This method provides a more comprehensive assessment of simplicity and aligns closely with human judgments of text simplification quality, particularly in readability and conciseness.

Together, BLEU and SARI provide a balanced perspective on output quality, with BLEU focusing on grammatical and semantic adequacy, and SARI addressing the transformation required for simplification.

## 7. Experiments and results

To measure the credibility of our corpus for automatic simplification, we built a cascade of phrase-based simplification systems using the original simplification S1 and expanded simplification variants (S2,S3,S4) (see section 2.6). Table 10 shows the corpus combinations and their corresponding BLUE and SARI scores.

**Table 10:** Blue and Sari scores of all models, Syntactic Simplification and Expanded Simplification Variant based models are shown in italic.

				Combined test-set		Self test-set	
	Simplification	Combination	Sentences	BLUE	SARI	BLUE	SARI
M1	*Syntactic (SS)*	*S1*	1200	30.3	40.8	36.0	39.0
M2	*SS* + ESV1	*S1* + S2.	2400	37.5	38.1	37.5	38.3
M3	*SS* + ESV2	*S1* + S3.	2400	39.6	30.4	37.6	30.2
M4	*SS* + ESV3	*S1* + S4.	2400	33.2	28.1	34.9	28.7
M5	ESV	S2 + S3 + S4.	3600	41.6	**47.5**	33.6	44.8
M6	*SS* + ESV	*S1* + S2 + S3 + S4.	4800	**59.6**	45.3	**59.6**	**45.3**

Table 10 details our experiments with different combinations of the simplification corpora. The first model M1 is built using the corpus and our initial syntactic simplification scheme and achieve the BLEU score of 30.3 and SARI score of 40.8. The concatenation of S1 and S3 shows 37.5 BLEU score and for the other concatenation of data results shown in. Our corpus may not be sufficient to build successful models but its useful to test generalization of model for simplification of sentences.

The results of the models shown in Table 10 indicate that the performance of the model improves as the size of the data increases. Specifically, the *S1* + S2 + S3 + S4 model, which is the largest corpus, achieves the highest BLEU score of 59.6. Similarly, in the self-references, the *S1* + S2 + S3 + S4 model also achieves the highest BLEU and SARI scores of 59.6 and 45.3, respectively. These findings suggest that the size of the corpus is a significant factor in the performance of the SMT-based simplification approach. However, in some cases, the use of multiple references can lead to a lower score for the model, as is the case with the SS + LS model trained on S1 + S4. This could be due to the fact that the model is generating simplified sentences that are different from the available reference sentences, but are still valid simplifications. In such cases, it is important to manually evaluate the output of the model to ensure that it is generating high-quality simplifications.

The results of SS + LS model demonstrate the potential of using SMT-based approaches for text simplification and provide a strong justification for the use of syntactic simplification techniques in this context. However, in some cases, the use of multiple references can lead to a lower score for the model, as is the case with the SS + LS model trained on S1 + S4. This could be due to the fact that the model is generating simplified sentences that are different from the available reference sentences but are still valid simplifications. In such cases, it is important to manually evaluate the output of the model to ensure that it is generating high-quality simplifications (Table 11).

**Table 11:** Comparison of output of all systems M1- M6 shows sentences generated by models and reference sentence presents mannualy simplification.

No.	System	Sentence
1	Original	آپنے گھرو کے پچھوکڑ پاروں فِلماں وِچ کم کرن اپڑی
	M1	آپنے گھروکے پچھوکڑ فِلماں وچ کم کرن پہنچ سکے
	M2	آپنے گھروکے پچھوکڑ پاروں فِلماں وِچ کم کرن اپڑی
	M3	آپنےھروکے پچھوکڑ پاروں فِلماں وِچ کم کرن اپڑی
	M4	آپنے نا گھر چھڈ کے فلماں وچ کم کرن
	M5	آپنے نا گھر چھڈ کے فلماں وچ کم کرن
	M6	آپنے گھر چھڈ کے فلماں وچ کم کرن اپڑی
	Reference	اپنا گھر چھڈ کے فلماں وچ کم کرن ائ سی
	English	She had left her home to work in films.
2	Original	پار وسدے باقی ویر جہڑے اکھاں توں اولھے پر لکھتاں راہیں دلاں وچ وسدے سن
	M1	گتوں پار وسدے باقی ویر جہڑے اکھاں توں اولھے پر دل دے نہڑے وسدے سن
	M2	وسدے باقی ویر جہڑے اکھاں توں اولھے پر دل دے نہڑے وسدے
	M3	اگتوں پار وسدے ویر اکھاں توں اولھے پر لکھتاں راہیں دلاں وچ وسدے سن
	M4	جہڑے اگتوں پار وسدے ویر اکھاں توں اولھے پر لکھتاں راہیں وچ وسدے سن
	M5	اگتوں پار وسدے ویر اکھاں توں اولھے پر لکھتاں راہیں وچ وسدے سن
	M6	پار وسدے باقی ویر جہڑے اکھاں توں اولھے پر لکھتاں راہیں دلاں وچ وسدے سن
	Reference	سدے باقی ویر جہڑے اکھاں توں اولھے پر لکھتاں راہیں وچ وسدے سن
	English	Settled remaining brothers who were out of sight but lived in hearts through.
3	Original	و ہی چونترے وچ بہہ کے کھان والیاں نوں وکھو وکھ کر ہی دتا
	M1	کو ہی چونترI پانڈے وچ کھان والیاں نوں وکھو وکھ ہو گۓ
	M2	چونترے وچ بہہ کے کھان والیاں نوں وکھو وکھ کر ہی دتا
	M3	چونترے وچ بہہ کے کھان والیاں نوں وکھو وکھ کر ہی دتا
	M4	اکو ہی چونترے وچ بہہ کے کھان والیاں نوں وکھو وکھ کر ہی دتا
	M5	اکو ہی چونترے وچ بہہ کے کھان والیاں نوں وکھو وکھ کر ہی دتا
	M6	اکو ہی پانڈے وچ بہہ کے کھان والیاں نوں وکھو وکھ کر ہی دتا
	Reference	اکو ہی پانڈے وچ بہہ کے کھان والیاں نوں وکھو وکھ کر دتا
	English	Those eating from the same pot were separated from each other.

In the first sentence,Model M1 substituted the word “پاروں” with “پہنچ سکے,” M5 and M6 introduced lexical substitutions like “چھڈ کے” and “نا گھر چھڈ کے” with M6 closely aligning with the reference sentence by emphasizing the key aspect of “leaving home to work in films. In the second sentence, M1 replaced “پاروں” with “تو” to express the causal relationship in simpler terms, aligning with the original intent but modifying the structure. M4 made a minor lexical substitution by replacing “پاروں” with “تو” preserving meaning. M5 and M6 substitute phrases like “پيداہون دے کارن” and “رکھيا سی” M6 aligned most closely with the reference sentence by accurately conveying the meaning of being named due to being born in an old Punjabi village.

## 8. Conclusion

This paper is committed to facilitating research on Punjabi sentence simplification. PUSIM, a new dataset for the evaluation of Punjabi SS models. The simplifications in PUSIM were written manually by human annotators, and the simplification operations are also labeled. An in-depth analysis of PUSIM includes word insertion, deletion, substitution, rearrangement, and rephrasing, each applied independently to assess its individual impact on readability. Through careful evaluation using automated reading evaluations, it has been shown that this method works, and steps towards making the Punjabi Shahmukhi writing system less complicated also have been made. Our central hypothesis—that a larger and more diverse training corpus would lead to better simplification models—was partially proven. The results strongly confirm that corpus size is a dominant factor in achieving high fluency, as evidenced by the best-performing M6 model which leveraged the entire. However, our findings also reveal that the quality and nature of the data are equally critical. Additionally, this explanation shows how careful research methods were and highlights the clarity and precision with which this simplification means was implemented from the outset.

Future research directions include expanding PUSIM with more diverse sentence structures, developing more sophisticated simplification models, and investigating the impact of different simplification strategies on various linguistic factors. This study provides a foundational dataset and a benchmark for future work in Punjabi text simplification. It establishes that effective simplification requires a careful balance between data quantity and the consistent application of simplification rules. The insights gained are a crucial step toward developing automated tools to enhance readability and accessibility for Punjabi readers.

## 8. Limitations

- Simplification Scope: Our simplification strategies focused primarily on lexical and syntactic complexity. We did not explicitly address more complex semantic simplifications, such as breaking down long concepts, adding explanations, or altering discourse structures.
- Generalizability of Methods: The syntactic simplification rules and ESV strategies were developed for a specific dataset. Their effectiveness and general applicability to any arbitrary Punjabi text require further validation.
- Our findings are therefore a baseline, and future work would benefit from applying more modern techniques with larger datasets.
